# Using statistical methods and genotyping to detect tuberculosis outbreaks

**DOI:** 10.1186/1476-072X-12-15

**Published:** 2013-03-16

**Authors:** J Steve Kammerer, Nong Shang, Sandy P Althomsons, Maryam B Haddad, Juliana Grant, Thomas R Navin

**Affiliations:** 1 Division of Tuberculosis Elimination, Centers for Disease Control and Prevention, 1600 Clifton Rd NE, Atlanta, GA, 30333, USA; 2 Northrop Grumman Corporation, 2800 Century Parkway NE, Atlanta, GA, 30345, USA

**Keywords:** Tuberculosis, SaTScan, Outbreak detection, Genotyping, Log-likelihood ratio, Cumulative sums

## Abstract

**Background:**

Early identification of outbreaks remains a key component in continuing to reduce the burden of infectious disease in the United States. Previous studies have applied statistical methods to detect unexpected cases of disease in space or time. The objectives of our study were to assess the ability and timeliness of three spatio-temporal methods to detect known outbreaks of tuberculosis.

**Methods:**

We used routinely available molecular and surveillance data to retrospectively assess the effectiveness of three statistical methods in detecting tuberculosis outbreaks: county-based log-likelihood ratio, cumulative sums, and a spatial scan statistic.

**Results:**

Our methods identified 8 of the 9 outbreaks, and 6 outbreaks would have been identified 1–52 months (median = 10 months) before local public health authorities identified them. Assuming no delays in data availability, 46 (59.7%) of the 77 patients in the 9 outbreaks were identified after our statistical methods would have detected the outbreak but before local public health authorities became aware of the problem.

**Conclusions:**

Statistical methods, when applied retrospectively to routinely collected tuberculosis data, can successfully detect known outbreaks, potentially months before local public health authorities become aware of the problem. The three methods showed similar results; no single method was clearly superior to the other two. Further study to elucidate the performance of these methods in detecting tuberculosis outbreaks will be done in a prospective analysis.

## Background

Statistical algorithms applied to disease surveillance data aim to identify which cases most likely represent outbreaks, before local public health authorities would otherwise become aware of them. Early detection of outbreaks may increase the ability of local authorities to prevent additional outbreak-related cases. The algorithms work by applying statistical techniques to reported cases of disease, laboratory data, or pharmacy data to identify unusual deviations from expected values; some techniques use historic data to detect deviations from temporal trends and others examine spatial or spatio-temporal differences in disease concentrations [1,2]. Non-statistical methods may also be applied to detect changes in the spatio-temporal distribution of disease burden.

Tuberculosis (TB) incidence has been declining in the United States for almost two decades, resulting in 10,528 reported cases and a rate of 3.4 per 100,000 in 2011 (5.8% and 6.4% decrease, respectively, from 2010) [3]. Molecular characterization of *Mycobacterium tuberculosis* isolates (TB genotyping) provides a mechanism to detect TB transmission events that might otherwise go unnoticed, based on the principle that epidemiologically linked cases share a similar genotype. In the United States, routine genotyping of *M. tuberculosis* isolates from culture-positive TB cases started in 2004 through the Centers for Disease Control and Prevention’s (CDC) National Tuberculosis Genotyping Service [4]. In 2010, CDC launched the TB Genotyping Information Management System (TB GIMS), a secure web-based database available to all 50 states, the District of Columbia, Puerto Rico, and the U.S.-affiliated Pacific Islands [5]. In 2008 and 2009, 80% and 87% of U.S. culture-positive cases, respectively, had complete genotype and surveillance records available in TB GIMS.

We retrospectively assessed the effectiveness of three statistical methods for detecting infectious disease outbreaks, using surveillance and genotyping data associated with TB outbreaks investigated by CDC during 2008–2009. We selected cumulative sums (CUSUM) as one of our outbreak detection methods since they are an industrial standard and used by CDC in the Early Aberration Reporting System [2]; however, CUSUM is more challenging to implement with rare diseases such as TB. SaTScan and county-based log-likelihood ratio (LLR) both use the likelihood ratio method, with SaTScan adding a geographic search function [6]. We included county-based LLR as one of our methods for comparison because it is simple and consistent with how TB control is structured in the U.S., and thus may be easily applied in our setting.

## Methods

### Data sources and definitions

We selected known TB outbreaks to compare the sensitivity of the three statistical algorithms. State and local health departments requested CDC assistance with nine TB outbreaks during 2008–2009; field investigations of individual patients and their epidemiologic links confirmed that these cases represented recent transmission and should be considered outbreaks [7]. The date that local public health authorities first became aware of the potential TB outbreak was typically several weeks, and occasionally months, before CDC was invited to assist; the best estimate of that date was therefore determined based on discussion with local authorities once CDC became involved.

Cases were considered clustered if their isolates exactly matched by both spoligotype and 12-locus Mycobacterial Interspersed Repetitive Unit genotype results [8]. Analyses performed using the selected three statistical methods were restricted to TB cases reported to the National TB Surveillance System (NTSS) after January 1, 2005, and before the outbreak investigation commenced, and to records with both a valid genotype and corresponding NTSS record in TB GIMS. Outbreaks investigated before 2008 were excluded due to insufficient background data, because 2005 is the earliest year with nationally representative genotyping data. Outbreaks investigated after 2009 were excluded because surveillance data were incomplete at the time of this analysis. The date used for all analyses was the count date available from NTSS data, defined as the date that the health department verified the case as TB and included it in the official case count.

Location data available from NTSS include the county (used for CUSUM and county-based LLR methods) and 5-digit zip code (used for SaTScan method) associated with the patient’s residence at the time of diagnosis as determined by the local health department. Home addresses are not collected by the surveillance system.

### Statistical methods

We assessed three methodologies: county-based log-likelihood ratio (LLR), cumulative sums (CUSUM), and a spatial scan statistic (SaTScan) (Table 1). CUSUM uses a temporal approach within a specified geographic area; county-based LLR and SaTScan both use spatial methods. The three detection methods were evaluated separately for the ability to detect each outbreak-associated genotype.

**Table 1:** Three statistical methods for detection of tuberculosis outbreaks, 2008-2009

**Method**	**Description**	**Geographic unit**	**Time period**	**Parameters for alert threshold**
County-based LLR (current TB GIMS method)	Measures difference between the observed and expected geospatial concentration of cases	County	2-year window and 3-year window	Currently designated in TB GIMS as a county-based LLR of ≥ 5
CUSUM	Calculates a monthly cumulative sum of variations between the observed and expected case counts; indicates an aberrant event above a threshold	County	Cumulative by month; 2-year moving window used to determine background rate	Threshold determined by background rate (based on 6^th^ lowest case count among all 2 year windows), alternative rate (background rate + 3), null average run length (expect, on average, one false alarm every 100 months)
SaTScan	Applies spatial scan statistic to determine areas with significantly higher geospatial concentration of cases	None assumed; a circular area is flexibly determined by algorithm; cases aggregated by zip code	2-year window and 3-year window	*P* value to determine location of clusters (< 0.05) using maximum search radius of 20, 50, and 100 km for the circular scanning

Definition of abbreviations: *CUSUM* = cumulative sums; *LLR* = log-likelihood ratio; *TB GIMS* = Tuberculosis Genotyping Information Management System.

#### Method 1: county-based LLR

The LLR method assumed cases follow a Poisson distribution, and compared the ratio of cases with the outbreak genotype to all genotyped TB cases inside a given geographic area, which we defined as a county, to the ratio in the rest of the United States. The LLR calculation is as follows:

$$\begin{array}{l} \mathrm{LLR}=\left[n_{i}*\text{log}\left(\frac{n_{i}/\left(n_{i}+N_{i}\right)}{n_{\mathrm{all}}/N_{\mathrm{all}}}\right)\right]\\ \;+\left[n_{o}*\text{log}\left(\frac{n_{o}/\left(n_{o}+N_{o}\right)}{n_{\mathrm{all}}/N_{\mathrm{all}}}\right)\right] \end{array}$$

where *n*_*i*_ = TB cases with the outbreak genotype inside the county, *N*_*i*_ = cases with all other genotypes inside the county, *n*_*o*_ = cases with the outbreak genotype outside the county, *N*_*o*_ = cases with all other genotypes outside the county, *n*_*all*_ = all cases with the outbreak genotype in the United States (i.e., *n*_*i*_ + *n*_*o*_), and *N*_*all*_ = all genotyped cases in the United States (i.e., *n*_*i*_ + *n*_*o*_ + *N*_*i*_ + *N*_*o*_).

A higher value of LLR indicates a greater likelihood that the specified county has an unexpected concentration of cases with a certain genotype. TB GIMS, which currently employs this methodology using a 3-year time window, reports the county-based LLR as an “alert” if a TB genotype in that county has an LLR ≥5; this threshold was selected by a group of national experts based on a review of TB genotyping clusters in the United States in 2009.

For each of the nine outbreaks, a county-based LLR was retrospectively calculated using both 2-year and 3-year moving time windows. The earliest time window had a start date of January 1, 2005; the window was then advanced 1 month at a time and the LLR was calculated in sequence. We determined whether and when each outbreak would have first alerted using the TB GIMS cut point.

#### Method 2: county-based CUSUM

CUSUM is used to detect temporal aberrations in a fixed geographic region, which we defined as a county. For each of the nine outbreak counties, we applied a Poisson distribution-based CUSUM procedure to the monthly number of TB cases with the outbreak genotype. The procedure carried out monthly statistical tests sequentially, with the null hypothesis defined as no aberration and the alternative hypothesis as the occurrence of some aberration.

A CUSUM curve was constructed with monthly updated values of the CUSUM statistic, and a horizontal line representing the critical value for rejecting the null hypothesis (threshold) was added to the graph. An aberration was noted at the month and year the CUSUM curve crossed above the threshold. Constructing the CUSUM curve and the threshold line requires specification of the following parameters: the background event rate (no aberration); a definition of the aberration that we would like to detect (in terms of increased event rate over the background rate); and the null average run length, defined as the acceptable false alarm rate (average number of months between two alarms when there is no aberration).

We counted the number of cases with the outbreak genotype within all 2-year time windows contained in the study period (i.e., with start dates of January 1, 2005 through January 1, 2008, advancing one month at a time). The case counts from the resulting 37 time windows were sorted and the sixth smallest count, determined heuristically, was used as the background rate; a rate of 0.5 was used if the count was zero. The aberrant rate was then defined as the background rate plus the minimal detectable outbreak. Based on CDC experience with previous TB outbreaks, we defined the minimal detectable outbreak as 3 cases in a 2-year period [7].

The threshold was chosen based on the background and aberrant event rates and a null average run length of approximately 100 months [9]. An alert was identified if and when the monthly CUSUM value exceeded the threshold.

#### Method 3: SaTScan

The spatial scan statistic, as implemented in the SaTScan software, has been used extensively to detect localized clusters of disease [10-14]. We applied SaTScan version 8.0.2 (Kulldorff, Boston) using a retrospective, purely spatial analysis and the discrete Poisson probability model. A detailed description of SaTScan methods can be found elsewhere [6]. Briefly, the LLR for the Poisson model is calculated for a pool of candidate circular windows with different locations and sizes. The spatial scan statistic is defined as the maximum LLR among all the candidate clusters, and is tested for statistical significance using 999 Monte Carlo replications to derive the *P* value, based on the distribution of the spatial scan statistic under the null hypothesis of spatial randomness of cases. After determining the most likely cluster (i.e., maximum SaTScan LLR), the algorithm continues to search for additional clusters under the assumption that a case can only be included in one cluster.

For our application, the SaTScan LLR compared the number of cases with the outbreak genotype inside the circle with the number outside the circle, searching for areas with high rates, under the Poisson probability model. We aggregated cases for each outbreak genotype by zip code as the geographic unit of analysis and defined all genotyped TB cases as the population at risk for having a specific outbreak genotype, also aggregated by zip code. Zip code centroid coordinates were obtained from Esri (Redlands, CA).

We ran each outbreak genotype multiple times using a maximum circle radius of 20, 50, and 100 km; we also examined both 2- and 3-year moving time windows with the same procedure used for the county-based LLR method. An alert was identified if and when the SaTScan cluster first became significant (*P* < 0.05).

### Application of aberration detection results

We determined whether and when each method detected the outbreak and compared it to the date that local public health authorities first reported becoming aware of the potential outbreak. To estimate the true impact for each outbreak, we included all cases during 2005–2009 that were confirmed by field investigations to be part of the outbreak, regardless of whether a genotype was available (e.g., culture-negative pediatric cases that were epidemiologically linked to a confirmed case) [7]. To estimate the number of potentially avertable cases in each outbreak, we calculated the number of outbreak cases occurring after the earliest date of detection by any method, had the retrospectively applied statistical alert methods been in place at that time.

#### Ethical review

Data used in this study were collected as part of routine disease surveillance and control activities and were not considered to constitute human subjects research requiring institutional review board approval.

## Results

### Effectiveness and timeliness of statistical methods

The retrospectively applied methods would have successfully identified six of the nine outbreaks before they were locally identified as a problem by the local public health authorities (Table 2). These statistical methods, when taken together, would have issued an alert 1–52 months (median = 10 months) before the local public health authorities identified a problem.

**Table 2:** Timeliness in detection of nine known tuberculosis outbreaks by local authorities and statistical method

**Outbreak**	**Spoligotype**	**MIRU**	**Earliest date of detection**				**Earliest detection method**
			**Local authorities**^**a**^	**County-based LLR alert **^**b**^	**CUSUM alert**	**SaTScan alert **^**c**^	
A	777776777760601	224325143323	05/04/09	07/31/07	06/30/07	07/31/07	CUSUM
B	700036777760771	222325133223	05/05/09	06/30/08	06/30/08	06/30/08	LLR, CUSUM, and SaTScan
C	740777607760771	223315193323	06/12/09	05/31/09	04/30/09	04/30/09	CUSUM and SaTScan
D	000000000003771	223325173533	06/01/08	None	06/30/07	None	CUSUM
E	676177607760771	224326153323	07/01/08	08/31/08	08/31/08	08/31/08	Local authorities
F	770000770000000	224125153322	08/01/09	06/30/09	06/30/09	06/30/09	LLR, CUSUM, and SaTScan
G	777776770000000	225325133324	02/07/08	03/31/08	04/30/08	03/31/08	Local authorities
H	777776757760771	223325143324	09/01/09	12/31/06^d^	04/30/05	12/31/06^d^	CUSUM
I	477777777720771	227325153323	08/28/09	None	None	None	Local authorities

Definition of abbreviations: *CUSUM* = cumulative sums; *LLR* = log-likelihood ratio; *MIRU* = Mycobacterial Interspersed Repetitive Units; *TB GIMS* = Tuberculosis Genotyping Information Management System.

^a^ Date that local public health authorities first noticed a problem.

^b^ Using a 2-year time window.

^c^ Using a 2-year time window and a 50 km maximum search radius.

^d^ Earliest possible date of alert is 12/31/06 (genotyping data in TB GIMS incomplete before 01/01/05).

Outbreak “D,” identified by local public health authorities 11 months after the time it was detected by CUSUM, was confirmed to be a true outbreak but was not detected by either county-based LLR or SaTScan; this outbreak’s genotype is the most commonly found genotype in the United States. Outbreaks “E” and “G” were detected by local authorities approximately 2 months before being detected by an aberration detection method. Outbreak “I” was not detected by any of our methods, and is an example of a widely distributed outbreak, involving 4 cases spread over 3 states and 4 different counties.

### Time period

Of seven outbreaks detected using the county-based LLR method, one alerted 1 month earlier when using a 2-year time period versus a 3-year period for calculating the county-based LLR, four alerted at the same time, and two were not comparable due to data not being available before 2005 (data not shown). The time window used for the SaTScan method had no effect on the timing of alerts; of seven outbreaks alerted by SaTScan with a 3-year window, five alerted at the same time when using a 2-year window, and the remaining two outbreaks were not comparable due to data not being available prior to 2005. Based on these results, we present subsequent data using a 2-year window.

Time period was not an issue for CUSUM as it detects temporal changes continuously using all cumulated data.

### SaTScan radius

When compared to a 50 km radius, running SaTScan with a maximum search radius of 20 km and 100 km had no substantial effect on the radius of the circle that defined the cluster, the LLR, or the number of outbreak cases included in the cluster for four of six alerted outbreaks examined (data not shown). One outbreak was split into two separate SaTScan clusters when a 20 km maximum search radius was used and one of the outbreak cases was excluded; for this outbreak the SaTScan analyses with the 50 km and 100 km maximum radius correctly grouped all the outbreak cases into one SaTScan cluster with the same circle radius and LLR. One outbreak had the same results for the 50 km and 100 km runs; however the 20 km analysis had a smaller radius defining the cluster (18.9 km vs. 36.2 km) and a slightly lower LLR, but all outbreak cases were still captured. Based on these results, we present subsequent SaTScan analyses using a 50 km maximum search radius.

### Performance of the three statistical methods

#### Method 1: county-based LLR

Seven of the nine outbreaks would have alerted using the county-based LLR method and a 2-year time window. Four would have alerted 1–32 months before the local public health authorities recognized the outbreak (median = 16 months earlier). Of the three remaining outbreaks, the county-based LLR would have alerted 2 months afterward for two and at approximately the same time for another.

#### Method 2: county-based CUSUM

Eight of the nine outbreaks would have been detected by CUSUM. For six, CUSUM would have alerted 1–52 months before local recognition of the outbreak (median = 10 months earlier). For the two remaining outbreaks CUSUM would have alerted approximately 2–3 months afterward.

Compared with the timing of the county-based LLR, CUSUM would have alerted 1 month earlier for outbreaks “A” and “C,” and 1 month later for outbreak “G”; three outbreaks would have alerted at the same time. For the remaining outbreak (i.e., outbreak “H”), results were not comparable because the earliest date of alert for the county-based LLR method was limited by incomplete TB GIMS data before 2005. For the outbreak not detected by county-based LLR (i.e., outbreak “D”), CUSUM would have alerted 11 months before the local public health authorities detected the problem.

#### Method 3: SaTScan

Seven of the nine outbreaks would have raised an alert using the SaTScan method with a maximum 50 km search radius and a 2-year time window. For five, SaTScan would have alerted from 1 to 32 months before the local health department recognized the outbreak (median = 10 months earlier). SaTScan would have alerted about 2 months after the local health department for the other two outbreaks.

SaTScan raised alerts for the same seven outbreaks that would have been alerted using the county-based LLR method. Compared to the timing of alerts using county-based LLR, SaTScan would have alerted 1 month earlier for outbreak “C,” and the remaining six were the same, although results for outbreak “H” were not comparable because the earliest detection date was limited by incomplete TB GIMS data before 2005.

### Timeliness of the alerts

When our retrospectively applied alert methods were combined, 46 (59.7%) of the 77 outbreak patients (including both culture-positive and culture-negative cases) in the nine outbreaks occurred after the detection method would have signaled an alert but before the local public health authorities noticed the problem (Table 3). These results are based on an assumption of real-time data availability; this issue is addressed in detail in the discussion section.

**Table 3:** Tuberculosis cases occurring after outbreak detection by retrospectively applied statistical methods, 2008–2009

**Outbreak**	**Outbreak cases**^**a**^**, n**	**Cases occurring after detection**^**b**^**, n (%)**	**Outbreak duration, months**^**c**^	**County size, sq km**^**d**^	**County population**^**d**^
A	16	16^e^ (100)	9	290	865,000
B	13	10 (76.9)	14	200	926,000
C	5	3 (60.0)	3	150	905,000
D	7	3 (42.9)	8	280	532,000
E	3	0 (0)	43	280	21,000
F	5	0 (0)	3	210	178,000
G	8	0 (0)	26	3050	1,954,000
H	16	14 (87.5)	15	150	742,000
I	4	0 (0)	27	NA^f^	NA^f^
Total	77	46 (59.7)			

^a^ Culture-positive and culture-negative cases reported in 2005–2009, and confirmed to be part of the outbreak at the end of the Centers for Disease Control and Prevention investigation.

^b^ Confirmed outbreak cases that occurred after the earliest date of detection among the three retrospectively applied statistical methods, and before the local public health authorities first noticed the outbreak.

^c^ Computed using culture-positive and culture-negative cases reported in 2005–2009, and confirmed to be part of the outbreak at the end of the Centers for Disease Control and Prevention investigation.

^d^ From 2010 U.S. Census, county size rounded to the nearest 10 sq km, county population rounded to nearest thousand persons.

^e^ Outbreak A included earlier cases that were not contained in the outbreak cases list developed during the investigation.

^f^ Outbreak spans multiple counties.

Table 3 also includes the duration of each outbreak as well as a comparison of the geographical size and population of the counties where outbreaks occurred.

## Discussion

Our results demonstrate that the three statistical methods, when applied retrospectively to routinely collected TB data, can successfully detect known TB outbreaks, potentially months before local public health authorities became aware of the problem. Early detection of outbreaks allows more prompt intervention potentially averting additional cases.

The three statistical methods we compared (county-based LLR, CUSUM, and SaTScan) showed similar results and no single method was clearly superior. The methods were all, to some degree, based on geospatial concentration, and predictably did not identify outbreak “I” where the TB diagnoses occurred in three different states, even though *M. tuberculosis* transmission had occurred in a single workplace [15]. County-based LLR and SaTScan did not identify outbreak “D,” which was associated with the most common genotype in the United States, accounting for 1,077 (4.1%) of the 25,973 TB cases with a genotype result during 2008–2009. While county-based LLR and SaTScan have successfully identified other outbreaks associated with common genotypes (unpublished data), we do not yet have enough experience with CUSUM to know if it consistently performs better than county-based LLR and SaTScan with common genotypes. Recent improvements in our genotyping methods may also improve our ability to detect outbreaks involving common genotypes. In 2009, CDC expanded the routine panel for Mycobacterial Interspersed Repetitive Units genotyping from 12 to 24 loci. This additional discriminatory power should increase the specificity of our detection methods, particularly for common TB strains such as the one associated with outbreak “D.”

For other diseases with shorter incubation periods, studies that have evaluated methods to detect outbreaks or aberrant clusters of disease have generally been temporally focused, often using syndromic surveillance data [1,16-18]. Some researchers have applied quality control schemes, such as CUSUM charts or variations on the CUSUM method, to detect shifts in reported counts of health events [2,19-23]. Others have used time series methods such as the autoregressive integrated moving average (ARIMA) model when cases of a disease are cyclical in nature and have seasonal trends [2,24-29]. Both methodologies use baseline data to establish expected counts in a given time interval, where the baseline period is typically defined using historical data that are representative of usual patterns in disease counts and do not reflect outbreaks or unusual events. Given the low incidence of TB in the United States and even lower counts of cases with a specific genotype, in addition to the often years-long incubation period for TB disease, our application of CUSUM involved sparse data. The lack of genotyping data before 2005 limited our ability to use an historical period to define the baseline, which led to our use of multiple 2-year time windows to establish the CUSUM background rate; we plan on revisiting our determination of baselines when a longer period of genotyping data is available.

Our examination of geospatial statistical methods was based on the assumption that TB transmission is mostly local [30-32]. Both the county-based LLR and SaTScan look for an elevated concentration of cases with a genotype in a geographic area compared with the concentration of that genotype outside the area. SaTScan addresses the bias introduced by the county-based LLR when cases are aggregated using a predefined geographic area [33]. Sensitivity analyses using different values of the SaTScan maximum search radius suggested that a scanning radius of 50 km was sufficient to detect the geographically concentrated outbreaks in this study.

Our analysis has several important limitations. First, the success of aberration detection methods fundamentally relies on the completeness, quality, and timeliness of the underlying data. Regarding the issue of missing data, several investigations in the United States have concluded that TB case detection and reporting are excellent [34-36]. All three methods use genotype data and genotyping is only possible on culture-positive TB cases, or approximately three quarters of TB cases reported to CDC [3]; 89% of culture-positive TB cases in 2009 had isolates submitted for genotyping. The omission of TB patients with culture-positive TB who do not have a genotyped isolate as well as culture negative cases may affect the performance of our methods in terms of detecting outbreaks and timeliness of the alerts. Of note, we captured 78% of the cases confirmed to be part of the nine outbreaks (data not shown).

With respect to timeliness our retrospective analysis assumed no delays in reporting; however, delays in data availability are inevitable, and real-life performance of our outbreak detection methods will be less robust. The median time between specimen collection and availability of both genotyping and surveillance data for a patient was 142 days when TB GIMS debuted in 2010 [37]. Factoring in a delay of 142 days (a delay anticipated to decrease as TB GIMS use increases), our statistical methods would have issued an alert before local public health workers noticed the problem for four of the nine outbreaks.

Second, as demonstrated by outbreak “I,” our methods were only designed to detect outbreaks in geographically limited areas. CUSUM or a purely temporal scan statistic could potentially detect geographically dispersed outbreaks if applied on a national or regional level. Third, we examined outbreaks that were investigated by CDC, and our results may not be generalizable to all U.S. TB outbreaks.

Fourth, our analysis used known outbreaks to examine the sensitivity and timeliness, but not specificity, of the statistical methods studied. During the 2-year period 2009–2010 only 282 county-based clusters met the alert threshold (LLR≥5) used in this paper and 590 SaTScan clusters met the threshold of p<.05, but the proportion of these clusters that represent false-positive alerts is unknown. Our focus in this study was the sensitivity of our methods in retrospectively detecting outbreaks and is an important first step in the assessment of the value of statistical outbreak detection; the next step is an examination of specificity. In a recent study local health authorities investigated 24 TB clusters of 6 cases or more to determine which ones were outbreaks; an algorithm using the SaTscan method had a specificity of 83.3% (5 of 6 clusters determined not to be outbreaks were not alerted using the algorithm) [38].

Finally, the number of cases that occurred after the alerts and before detection by local authorities that could have been prevented is indeterminate.

In conclusion, the best future application of these statistical methods to outbreak detection might be to use all three approaches, while gaining experience about the relative uncertainty associated with each method’s performance and applying this experience to improve model accuracy. Determining how to interpret discrepant results and exploring how to optimally set model input parameters will be important steps as well. We are now prospectively examining the performance of these methods in detecting TB outbreaks.

## Abbreviations

CDC: Centers for Disease Control and Prevention; CUSUM: Cumulative sums; LLR: Log-likelihood ratio; TB: Tuberculosis; TB GIMS: Tuberculosis Genotyping Information Management System.

## Competing interests

The authors declare that they have no competing interests.

## Authors’ contributions

JG, TRN, and JSK provided the conception and design for the study. JSK, SA, MBH extracted and collected the data. NS, SA, JSK, MBH conducted the analyses and interpretation of results. JSK wrote the manuscript. JG and TRN provided overall study supervision. All authors participated in the review and approval of the manuscript.
